# Assessing Medical Students’ Ophthalmology Exposure and Its Role in Their Speciality Choices: A National Survey

**DOI:** 10.7759/cureus.90814

**Published:** 2025-08-23

**Authors:** Maiar Elhariry, Amr Badawy, Aymen Mustafa, Ala Bashir, Mohammed Athif Khan, Sai Sirikonda, Akash Doshi

**Affiliations:** 1 Ophthalmology, Midlands Metropolitan University Hospital, Smethwick, GBR; 2 General Surgery, Midlands Metropolitan University Hospital, Smethwick, GBR; 3 Trauma Surgery, Midlands Metropolitan University Hospital, Smethwick, GBR; 4 Medicine, Worcestershire Acute Hospital NHS Trust, Worcester, GBR; 5 Medicine, Mersey and West Lancashire Teaching Hospitals NHS Trust, Prescot, GBR; 6 Medicine, Barts Health NHS Trust, London, GBR

**Keywords:** medical education, ophthalmology, ophthalmology residency, ophthalmology teaching, opthalmology exposure

## Abstract

Introduction

Ophthalmology remains a competitive speciality requiring early and structured exposure to influence medical students' interest and preparedness. Despite its competitiveness, exposure to ophthalmology in the undergraduate curriculum is minimal, especially in comparison to other specialities. Exposure to ophthalmology, as well as other key factors during medical school and beyond, can affect students' decisions to pursue ophthalmology.

Aims

This study aimed to evaluate ophthalmology exposure among United Kingdom (UK) medical students and its influence on their likelihood of pursuing ophthalmology as a career. It also aimed to identify factors that can influence this decision.

Methods

A national cross-sectional survey was administered to final-year UK medical students attending ophthalmology-focused sessions hosted by Mind the Bleep (MTB), a national free educational platform, from December 2024 to April 2025. The survey assessed curricular coverage, teaching quality, alignment with United Kingdom Medical Licensing Assessment (UKMLA) standards, and the impact of these factors on career decision-making. Quantitative responses were analysed using IBM SPSS Statistics for Windows, Version 25 (Released 2017; IBM Corp., Armonk, New York, United States), while thematic analysis of free-text responses was conducted in NVivo.

Results

Of the 193 respondents, 176 UK-based students were included. Ophthalmology education as a whole was rated poorly in the domains of curricular coverage (mean 2.94/5), teaching support (mean 2.95/5), and alignment with learning needs (mean 2.96/5). A more detailed analysis of external factors influencing students’ career intentions revealed that both exposure to ophthalmology (mean 3.86/5, 0.95 Standard Deviation (SD)) and the perceived quality of teaching (mean 3.76/5, 0.95 SD) were positively associated with an increased likelihood of pursuing the speciality. Qualitative responses highlighted work-life balance, placement quality, and career prospects as major additional influences.

Conclusions

Undergraduate exposure to ophthalmology significantly impacts students’ speciality preferences, though current exposure is perceived as insufficient. Enhancing structured ophthalmology teaching and aligning curricula with national standards may encourage more students to consider the speciality. Broader educational reforms should incorporate lifestyle, mentorship, and placement experiences to support informed and equitable career decision-making.

## Introduction

Ophthalmology is a highly competitive speciality, with a competition ratio of 14.41 in 2024, and a complex field that demands both technical proficiency and a strong theoretical foundation [1]. The United Kingdom Medical Licensing Assessment (UKMLA) is a national examination taken by final-year medical students to demonstrate readiness for safe practice and is a requirement for graduation [2]. It highlights, amongst other things, key ophthalmology presentations that they must recognise and be familiar with managing. These include presentations like red eye, gradual changes or loss of vision, and specific diseases like acute and chronic glaucoma, diabetic eye disease, and uveitis [2]. Nevertheless, in most medical schools, ophthalmology is not a mandatory core rotation with limited teaching through clinical skills sessions or lectures compared to other specialities [3,4]. For aspiring ophthalmologists, much of this foundational knowledge is developed during medical school, making early and structured exposure critical to success [4,5]. Medical students often base their speciality decisions on their undergraduate experiences, and evidence suggests that comprehensive exposure to ophthalmology during this period significantly increases the likelihood of pursuing it as a career [1,6]. In addition to academic content, the educational environment and availability of mentorship play a pivotal role in shaping students’ career trajectories [6,7]. Early engagement with the speciality not only facilitates the development of essential clinical competencies but also helps students understand the broader professional and personal demands of a career [4]. As such, medical schools are uniquely positioned to influence speciality choices by designing curricula and offering extracurricular opportunities that provide meaningful exposure and support early career planning [8].

Despite the recognised importance of ophthalmology education in influencing career choices, there is a paucity of research specifically examining the impact of undergraduate medical curricula and enrichment opportunities offered on students’ decisions to pursue ophthalmology [3-5]. This gap in the literature underscores the need for targeted investigations into how curricular exposure shapes speciality selection [6,8]. Advancing research in this area is crucial for guiding curriculum development and ensuring that medical students are adequately supported in making informed and deliberate career choices [6-9].

This study aims to address the existing gap in the literature by exploring how exposure to ophthalmology during medical school influences students’ speciality choices. We examine the extent and quality of curricular coverage as well as other influential factors, such as teaching quality and the broader educational environment. Importantly, we recognise the need for medical school curricula to maintain a non-biased approach, ensuring that students are supported in exploring a diverse range of specialities without undue influence. By identifying how curricular structure and exposure shape career decision-making, this study seeks to inform strategies for enhancing undergraduate ophthalmology medical education, ultimately promoting more balanced, student-friendly exposure across specialities and enabling students to make well-informed, reflective career choices [5,6].

## Materials and methods

Data collection

A national survey was conducted as part of the registration process for the Mind the Bleep (MTB) Final Year Series. The MTB Final Year Series was an online national teaching programme aimed at final-year medical students in preparation for the UKMLA, which covered a variety of specialities, including ophthalmology. The final year ophthalmology series sessions ran from December 2024 to April 2025 and focused on five core topics aligned with the UKMLA finals curriculum (Table 1). As part of the registration for these sessions, students were required to complete a survey designed to capture data on their exposure to ophthalmology and their views on the quality of teaching they received in medical school. This approach ensured that the respondents were all final-year medical students, actively preparing for exams, and engaging with ophthalmology content relevant to their curriculum. This design was informed by previous research on educational surveys that utilise event-based registration to capture relevant data from participants [9,10]. 

**Table 1:** Topics taught as part of the ophthalmology teaching series.

Date	Topics
17/12/2024	Ophthalmology 101: anatomy & eye history
14/01/2025	Visual pathways, Visual field defects & eye exam
11/02/2025	Rapid review: quick-fire questions & image quiz
11/03/2025	Acute ophthalmology
08/04/2025	Chronic ophthalmology

Sessions and their respective registration forms were advertised via the MTB social media pages. Additionally, sessions were advertised via national universities’ final-year WhatsApp groups, shared by MTB representatives for each respective university. This approach was intended to maximise reach to final-year students and ensure equitable access for all students, including those not affiliated with ophthalmology-specific societies or speciality-focused educational groups who may have otherwise been unaware these teaching sessions were being offered.

Survey design and questions

The survey was designed to assess key aspects of medical students' exposure to ophthalmology and the role this had in influencing their speciality choices. The questions were grouped into four primary domains, each chosen to address factors that have been discovered to influence speciality choice in medical students in previous works [6-8].

The first area of assessment was the coverage of ophthalmology within the medical curriculum [3,4]. Students were asked to evaluate how well ophthalmology was covered in their medical school or trust's curriculum, as well as which topics within ophthalmology they felt were inadequately addressed. This section was designed to highlight any gaps in the ophthalmology curriculum, which could then inform future curriculum development. Research has shown that inadequate exposure to a speciality can negatively affect students' understanding and interest in pursuing that field [5,6].

The second area focused on the quality and availability of ophthalmology teaching [1]. Participants were asked to specify in which year(s) ophthalmology was taught, the types of teaching methods used (e.g., lectures, practical sessions, tutorials), and the amount of teaching they received. Additionally, students were asked whether their medical school or trust had tailored its ophthalmology teaching to align with the UKMLA framework and recent changes in medical education. This section aimed to measure both the quantity and quality of teaching, which has been shown to play a critical role in shaping students’ interest in a speciality [6].

The third section assessed the impact of ophthalmology teaching on career choices. Students were asked to rate the influence of their ophthalmology exposure during medical school on their likelihood to pursue ophthalmology as a speciality. Participants were also asked when they made their speciality decision and whether ophthalmology was a field they were considering. 

Finally, the survey included open-ended questions to capture additional factors that might influence students' decisions to pursue ophthalmology. Students were encouraged to share their thoughts on other considerations such as work-life balance, the competitiveness of the speciality, mentorship experiences, and the influence of positive or negative feedback from peers and faculty. This open-ended section was designed to gain deeper insight into the personal and professional factors that shape students' speciality choices, especially in the context of ophthalmology, as identified in previous research on medical career decision-making [11,12].

To aid students in better expressing the intensity of their feelings when answering particular questions and also allowing some data to be summarised and analysed statistically (e.g., calculating means, medians, or testing correlations), making it easier to interpret trends and patterns, Likert scales were used. In our Likert scales, 1 was synonymous with strongly negative feelings, whereas 5 was synonymous with strongly positive feelings, with 3 meaning no feelings towards it and 2 and 4 meaning mildly negative or positive feelings, respectively.

Data analysis

Data analysis was conducted using both quantitative and qualitative methods to ensure a comprehensive understanding of the factors influencing students' decisions regarding ophthalmology as a career. Quantitative data from Likert scale responses were analysed to assess students' perceptions of ophthalmology curriculum coverage, the quality of teaching, and its impact on their career decisions. Descriptive statistics, such as means and frequencies, were calculated to summarise the data. Qualitative data, obtained from open-ended responses, were analysed using thematic analysis. This allowed for the identification of recurring themes regarding the factors influencing students' career choices. This was done by two independent coders to minimise bias and promote generalisability. 

The data was analysed using IBM SPSS Statistics for Windows, Version 25 (Released 2017; IBM Corp., Armonk, New York, United States) for the quantitative responses and NVivo version 12 (QSR International, Melbourne, Australia) for the qualitative analysis.

## Results

A total of 193 students participated across five UK-based ophthalmology teaching sessions. Responses from 176 UK-based students were included in the analysis to ensure consistency with the UK undergraduate medical curriculum and the UK Medical Licensing Assessment (UKMLA) framework (Table 2). Responses from international students were excluded due to variations in curricular structures and assessment standards abroad when compared to the United Kingdom.

**Table 2:** The distribution of responses based on university from UK-based medical students.

Institution	Number of responses
University of Sheffield	22
Ulster University	17
University of Warwick	16
University of Birmingham	13
St George's, University of London	12
The University of Manchester	12
University of Dundee	11
University College London	11
Swansea University	11
Keele University	10
Exeter University	10
University of Cambridge	9
University of Oxford	9
University of Plymouth	7
University of Liverpool	7

Coverage of ophthalmology in the curriculum

Participants rated the question, “How well is this topic covered by your medical school teaching?”, about the individual UKMLA-aligned ophthalmology topics included in the teaching series. The mean rating was 2.94 (Standard Deviation [SD]: 0.96; range: 1-5) (Table 3). When asked, “How well is Ophthalmology covered by your medical school teaching?”, the average score was also 2.95 (SD: 0.96; range: 1-5) (Table 3). This suggests that ophthalmology remains insufficiently represented within the undergraduate curriculum, with a majority of students sharing more negative sentiments towards the current state of ophthalmology teaching at medical school. The relatively broad SD implies that student opinions were somewhat divided, with some perceiving better coverage than others.

**Table 3:** Summary of medical students’ ratings on ophthalmology teaching and its impact as per their responses to the questionnaire. UKMLA: United Kingdom Medical Licensing Assessment

Question	Mean Score	SD
Curriculum Coverage		
How well is each topic covered by your medical school teaching?	2.94	0.96
How well is ophthalmology overall covered by your medical school teaching?	2.95	0.96
Teaching Quality & Availability		
Awareness of UKMLA content and recent changes	3.01	0.93
Does your university provide adequate support and teaching?	2.95	0.88
Does your university/instructor cater to your learning style?	2.96	0.84
Impact on Career Choice		
Does your exposure to ophthalmology affect your likelihood of pursuing it?	3.86	0.95
Does teaching on ophthalmology affect your likelihood of pursuing it?	3.76	0.95

Teaching quality and availability

Students were asked to rate their awareness of UKMLA content and recent changes, resulting in a mean score of 3.01 (SD: 0.93; range: 1-5) (Table 3). When asked whether they felt their university provided adequate support and teaching, the average rating was 2.95 (SD: 0.88; range: 1-5) (Table 3). Similarly, the question regarding whether their university or instructors catered to their learning styles received a mean score of 2.96 (SD: 0.84; range: 1-5) (Table 3). These moderate scores suggest a neutral level of satisfaction with how well medical school education is tailored to students’ needs, potentially indicating a lack of strong engagement or enthusiasm with the current curriculum. The smaller SDs indicate fewer extreme opinions compared to the curriculum coverage domain, suggesting students share similar moderate views on teaching quality and availability.

Impact on career choice

The influence of exposure and teaching on speciality choice was consistently rated highly. In response to “Does your exposure to ophthalmology affect your likelihood to pursue it?”, the mean score was 3.86 (SD: 0.95; range: 1-5) (Table 3). For the question, “Does teaching on ophthalmology affect your likelihood to pursue it?”, the average was 3.76 (SD: 0.95; range: 1-5) (Table 3). The results indicate that both exposure to ophthalmology and teaching in the speciality have a moderately positive influence on students’ likelihood of pursuing ophthalmology as a career. With mean scores of 3.86 and 3.76, respectively, students generally perceive that their experiences and education in ophthalmology increase their interest in the field. Although these ratings are not at the highest end of the scale, they reflect a favourable impact, suggesting that improved or increased exposure and quality teaching could further enhance students’ motivation to consider ophthalmology as a speciality choice. 

The SD remains around 0.95, suggesting while students generally agree that ophthalmology exposure positively influences career decisions, there is still substantial variation among individuals. Some students may feel strongly influenced, whereas others are less so.

Other influencing factors

A total of 176 free-text responses were analysed in response to the question, “What other aspects might influence your likelihood to pursue a speciality?”. These responses indicate that, beyond teaching, lifestyle factors and the quality of clinical placements are key drivers in speciality decision-making. The top three common themes of factors influencing the participants’ decision to pursue Ophthalmology, as indicated by students’ responses, were: work-life balance (referenced by 60 respondents), placement experience (referenced by 53 respondents), and career prospects (referenced by 36 respondents). The most frequently cited factors are included in Table 4.

**Table 4:** Common themes of factors influencing students' likelihood to pursue ophthalmology.

Factor	Number of Mentions	Example Quotations from Answers
Work-life balance	60	“work-life balance”, “less likely to get called in out of hours”, “surgical but fewer on calls”
Placement experience	53	“placement”, “medschool allocation”, “ophthalmic team during placement”
Career prospects	36	“Sub-specialities”, “career”, “training”
Interest	20	“interest”, “interesting”, “curious”
Pay	18	“salary”, “rich”, “pay”
Training structure and security	18	“run-through”, “do not have to worry about a bottleneck”, “once you are in you are okay”
Ophthalmology trainees feedback	17	“trainees are happy”, “recommended I pursue it”
Research opportunities	9	“academic potential”, “academia”, “research”

## Discussion

Ophthalmology remains one of the most sought-after specialties in medicine with a high ratio of applicants to training places available. This national survey offers important insights into the state of undergraduate ophthalmology education in the UK and its influence on medical students’ speciality choices. Despite ophthalmology being one of the most competitive specialities, with a competition ratio of 14.41 in 2024 [13], the authors’ findings suggest that exposure to ophthalmology during undergraduate training remains limited and inconsistent across medical schools. The mean rating for ophthalmology curriculum coverage was 2.94 out of 5, with similar scores for teaching support (2.95) and alignment with students’ learning needs (2.96), reflecting a generally neutral or slightly negative perception of the current provision. This is consistent with previous national surveys, which have highlighted the underrepresentation of ophthalmology within UK medical curricula and the potential consequences for students’ understanding and interest in the speciality [3,4].

The quantitative analysis revealed that both exposure to ophthalmology (mean 3.86/5) and the perceived quality of teaching (mean 3.76/5) were positively associated with an increased likelihood of pursuing the speciality. These findings reinforce the importance of early and structured exposure in influencing career decisions. This echoes longitudinal research demonstrating that undergraduate teaching and clinical experiences are among the strongest predictors of speciality choice [8]. Notably, students also reported only moderate awareness of UKMLA content and recent changes (mean 3.01/5), suggesting a need for better alignment of undergraduate teaching with national standards and assessment frameworks [2]. The relatively broad standard deviations observed in ratings for curriculum coverage and teaching quality suggest variability in students’ experiences, likely reflecting differences in curriculum structure, teaching methods, and institutional priorities across UK medical schools.

The qualitative analysis of free-text responses provided a further dimension to these findings. Students identified work-life balance, the quality of clinical placements, and career prospects as the most influential factors in speciality decision-making, with work-life balance, placement experience and career prospects specifically referenced by a large proportion of respondents. Other important considerations included personal interest, pay, training structure, and feedback from current ophthalmology trainees. These results align with existing medical education literature, which emphasises the multifactorial nature of speciality choice and the interplay of both intrinsic and extrinsic motivators [12]. The importance of lifestyle considerations and placement experiences in particular suggests that interventions aimed at improving these aspects could have a substantial impact on the attractiveness of ophthalmology as a career. Another notable strength of this study is its national scope and focus on final-year students preparing for the UKMLA, ensuring that the findings are relevant to current curriculum standards and the realities of modern medical education. The mixed-methods approach, combining quantitative and qualitative data, allowed for a nuanced exploration of both the measurable impact of teaching and the complex, subjective factors that influence career decisions [14]. In developing the survey, the authors assessed multiple relevant dimensions, including curriculum coverage, teaching methods, timing, alignment with national standards, and the impact on career choice. Thematic analysis was employed for qualitative data, a widely accepted and flexible method for identifying patterns and themes in medical education research [15].

Some limitations should be acknowledged. The survey was distributed to students attending ophthalmology-focused teaching events, which may have introduced selection bias towards those already interested in the speciality [16]. This could mean that our findings overestimate the level of interest in ophthalmology and the perceived impact of teaching exposure. The reliance on self-reported data introduces the possibility of recall or response bias, as students may not accurately remember or may overstate their experiences [16]. The survey was distributed at 15 of the 44 medical schools in the UK, and of the 15 medical schools surveyed, some contributed a larger proportion of the respondents than others [17]. This could be another cause of selection bias, as some demographics may have been over- or under-represented. Lastly, the inclusion of only UK medical schools, though necessary for curriculum consistency, may limit the generalisability of the findings to the broader medical student population.

Based on these findings, the authors recommend several strategies for enhancing undergraduate ophthalmology education and supporting informed speciality choices. Medical schools may wish to prioritise early and structured exposure to ophthalmology, ensuring that core topics are adequately covered and that teaching aligns with UKMLA requirements. Integrating ophthalmology teaching into other specialities, such as neurology and primary care, and providing dedicated clinical placements could improve both knowledge and interest. Adopting varied and interactive teaching methods, such as simulation, case-based learning, and mentorship programmes, may further increase student engagement and satisfaction [1]. Faculty development initiatives should be implemented to ensure that teaching is responsive to students’ learning needs [18]. Additionally, medical schools and training bodies should highlight the work-life balance and career prospects in ophthalmology, and ensure that placements provide positive, supportive experiences. Opportunities for student involvement in research and academic ophthalmology should also be expanded, and access to career guidance and mentorship should be improved.

## Conclusions

This study highlights the critical influence of undergraduate exposure and the quality of teaching on medical students’ interest in pursuing ophthalmology. To foster informed and equitable speciality choices, medical schools should prioritise early, structured engagement with ophthalmology and refine teaching approaches to align with current curricular frameworks. In addition, future research should investigate the long-term outcomes of such educational interventions and explore how factors like mentorship, clinical placements, and institutional support further shape career decision-making within this competitive field.

## Appendices

Questionnaire

https://drive.google.com/file/d/1c777kv5XVTxEb1m36xkR_CpzrKtc9xR9/view?usp=sharing
